# Intermittent parathyroid hormone (1–34) supplementation of bone marrow stromal cell cultures may inhibit hypertrophy, but at the expense of chondrogenesis

**DOI:** 10.1186/s13287-020-01820-6

**Published:** 2020-07-29

**Authors:** Ena Music, Kathryn Futrega, James S. Palmer, Mackenzie Kinney, Bill Lott, Travis J. Klein, Michael R. Doran

**Affiliations:** 1 School of Biomedical Sciences, Faculty of Health, Queensland University of Technology (QUT), Brisbane, Australia; 2 Centre for Biomedical Technologies, Queensland University of Technology (QUT), Brisbane, Australia; 3 Translational Research Institute, Brisbane, Australia; 4 Institute of Health Biomedical Innovation (IHBI), Queensland University of Technology, Brisbane, Australia; 5 School of Mechanical, Medical and Process Engineering, Science and Engineering Faculty, Queensland University of Technology (QUT), Brisbane, Australia; 6 Mater Research Institute, Translational Research Institute (TRI), University of Queensland (UQ), Brisbane, Australia

**Keywords:** Mesenchymal stem cell, Chondrogenesis, Hypertrophy, Parathyroid hormone, Microwell

## Abstract

**Background:**

Bone marrow stromal cells (BMSC) have promise in cartilage tissue engineering, but for their potential to be fully realised, the propensity to undergo hypertrophy must be mitigated. The literature contains diverging reports on the effect of parathyroid hormone (PTH) on BMSC differentiation. Cartilage tissue models can be heterogeneous, confounding efforts to improve media formulations.

**Methods:**

Herein, we use a novel microwell platform (the *Microwell-mesh*) to manufacture hundreds of small-diameter homogeneous *micro*-pellets and use this high-resolution assay to quantify the influence of constant or intermittent PTH(1–34) medium supplementation on BMSC chondrogenesis and hypertrophy. *Micro*-pellets were manufactured from 5000 BMSC each and cultured in standard chondrogenic media supplemented with (1) no PTH, (2) intermittent PTH, or (3) constant PTH.

**Results:**

Relative to control chondrogenic cultures, BMSC *micro*-pellets exposed to intermittent PTH had reduced hypertrophic gene expression following 1 week of culture, but this was accompanied by a loss in chondrogenesis by the second week of culture. Constant PTH treatment was detrimental to chondrogenic culture.

**Conclusions:**

This study provides further clarity on the role of PTH on chondrogenic differentiation in vitro and suggests that while PTH may mitigate BMSC hypertrophy, it does so at the expense of chondrogenesis.

## Background

Articular cartilage is an avascular, aneural tissue with limited regenerative potential. This has motivated significant investment into cell-based therapies with potential to repair cartilage defects and delay or prevent osteoarthritis. Bone marrow stromal cells (BMSC, also known as mesenchymal stem cells) have emerged as a promising cell source for cartilage defect repair [1]. However, while BMSC appear to be able to form chondrocyte-like cells in vitro, these cells appear to engage an intrinsic endochondral differentiation program and yield mineralised hypertrophic tissue when implanted in vivo [2]. The search for compounds that mitigate BMSC hypertrophy is an active area of research, previously reviewed here [2, 3].

Parathyroid hormone (PTH) [4, 5] and its homologue, PTH-related protein (PTHrP) [6], are candidate molecules that may be useful to mitigate BMSC hypertrophy. PTH and PTHrP are expressed in different tissues, but share an amino-terminal sequence that binds and signals through a common G-protein coupled receptor, PTH/PTHrP type 1 receptor (PTH1R) [7]. As a result, an active 34-amino acid fragment of the PTH/PTHrP amino-terminus, PTH(1–34), is often used in studies. PTH1R is found on the surface of a variety of cell types including developing and mature chondrocytes and osteoblasts [8–10]. In previous studies with chondrocytes, PTH signalling was shown to increase type II collagen synthesis [11] and proteoglycan synthesis [11–16], while others, paradoxically, showed that PTH inhibited type II collagen synthesis [4, 17, 18]. In mesenchymal progenitor cell lines (C3H10T1/2), PTH appeared to play different roles during different stages of chondrogenesis, enhancing early chondrogenic differentiation while suppressing the late stages of chondrogenic maturation and osteogenesis [19]. In rats, PTH significantly increased aggrecan levels in chondrocytes isolated from day 17 and 18 embryos, but no increase was observed in chondrocytes harvested from embryos at day 20 or 21 of gestation [16]. These observations suggest that the stage of chondrogenesis may be important for PTH signalling outcomes.

PTH has been explored in BMSC differentiation cultures with varying results. Chen et al. reported that PTH-induced rabbit BMSC chondrogenesis resulted in better repair of articular cartilage injury in rabbits, compared with non-PTH-treated BMSC [20]. Zhang et al. observed a dose-dependent effect, whereby lower concentrations (1–10 nM) of PTH promoted BMSC chondrogenesis, while higher concentrations (> 100 nM) suppressed matrix production [18]. The timing of PTH dosing has also been reported to influence BMSC differentiation. In mildly adipogenic cultures, intermittent PTH treatment inhibited human BMSC adipogenesis, while constant PTH treatment did not [21]. Intermittent PTH treatment suppressed rat BMSC osteogenesis, while constant PTH treatment promoted osteogenesis [22]. Using a single injection in mice [6] and intermittent injection in humans [10], PTH was shown to improve osteogenic differentiation of BMSC, which may function through a mechanism that enhances BMP signalling [6]. In chondrogenic human BMSC pellet cultures, intermittent PTH treatment appeared to restore chondrogenic differentiation that was lost in cultures treated with constant PTH, also leading to reduced Indian hedgehog (IHH) expression and alkaline phosphatase (ALP) activity [23]. These findings suggest that a PTH treatment regimen may be important in controlling the differentiation of BMSC.

Traditional BMSC chondrogenic pellet cultures typically generate tissues that are physically large (2–3 mm diameter), causing steep diffusion gradients and result in heterogeneous tissue formation [24, 25]. Heterogeneous tissue can confound the study of subtle differences in differentiation outcomes [24, 25]. In these large diameter tissues, differentiation occurs at different rates depending on the spatial location of cells within the tissue. In previous work, we developed the *Microwell-mesh* [24], a high-throughput microwell platform that enables the manufacture of hundreds of homogeneous cartilage *micro*-pellets (sometimes referred to as microtissues) and maintains these *micro*-pellets in individual microwells over extended culture periods. As a result of their smaller size (0.5–1 mm diameter), relative to traditional pellet cultures, *micro*-pellets are more sensitive to differentiation signalling factors [26].

In this study, we used the *Microwell-mesh* platform to manufacture uniform BMSC *micro*-pellets and cultured them under three conditions: (1) control (chondrogenic medium with no PTH), (2) intermittent PTH (2.5 nM PTH(1–34), 6-h treatment every 48 h), and (3) constant PTH (2.5 nM PTH(1–34), continuous treatment, re-supplemented every 48 h). BMSC chondrogenesis was evaluated at 7 and 14 days by quantifying *micro*-pellet and medium glycosaminoglycan (GAG) content, *micro-*pellet histology, and chondrogenic/hypertrophic gene expression. Using this more sensitive *micro*-pellet culture approach, we aimed to clarify the influence of PTH on BMSC in vitro chondrogenesis.

## Methods

### BMSC isolation and culture

BMSC isolation and characterisation were performed as previously described [25]. Briefly, 20 mL of bone marrow aspirate was collected from the iliac crest of fully informed and consenting healthy adult donors. The collection was approved by the Mater Misericordiae Ltd. Human Research Ethics Committee (EC00332) and the Queensland University of Technology Human Ethics Committee (EC00171, 1000000938). The bone marrow aspirate was diluted 1:1 with 2 mM EDTA/PBS and overlaid onto 15 mL of Ficoll-Paque PLUS (GE Healthcare). The tubes were centrifuged for 30 min at 400×*g*, and interface cells were collected, washed, and resuspended in low glucose Dulbecco’s modified Eagle’s medium (LG-DMEM; Thermo Fisher Scientific) containing 10% fetal bovine serum (FBS; Thermo Fisher Scientific), 10 ng/mL fibroblast growth factor-1 (FGF-1; PeproTech), and 100 U/mL penicillin/streptomycin (PenStrep; Thermo Fisher Scientific). Cells were seeded in T175 cm^2^ flasks (Nunc) and incubated overnight in a 20% O_2_ and 5% CO_2_ incubator at 37 °C. Media were exchanged the following day to enrich for plastic-adherent cells. Cells were subsequently grown in 2% O_2_ and 5% CO_2_ at 37 °C. When the cells reached 80–90% confluence, they were passaged using 0.25% Trypsin/EDTA (Thermo Fisher Scientific) and re-seeded at 1500 cells/cm^2^. All experiments were carried out with three different BMSC donors, referred to as donors 1, 2, and 3.

### Fabrication and preparation of the *Microwell-Mesh* system

**Fig. 1:**
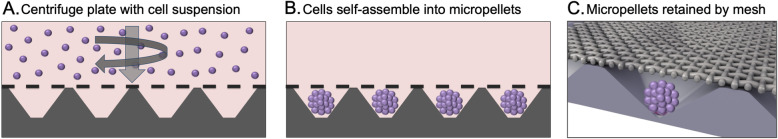
The *Microwell-mesh* enables rapid manufacture of hundreds of uniform *micro*-pellets, simultaneously. **a** Using a plate centrifuge, BMSC are pelleted through the porous mesh into individual microwells. **b** BMSC self-assemble into *micro*-pellets after a few hours of culture. **c** The nylon mesh retains individual *micro*-pellets in discrete microwells over the culture period. Image C was slightly modified from an image originally provided by abpLearning (medical-animations.com, Australia and [26]) using SoftImage (Autodesk, Montreal, Canada) and gifted to the Doran Laboratory

Figure 1 provides a schematic of how the *Microwell-mesh* enables the rapid manufacture and culture of hundreds of uniform *micro*-pellets. The process of fabricating the *Microwell-mesh* was detailed previously by our laboratory [24]. In brief, degassed polydimethylsiloxane polymer (PDMS, SYLGARD 184 Silicone Elastomer Kit; 10:1 polymer to crosslinker) was poured into a microwell mould and permitted to cure for 60 min at 80 °C. The resultant PDMS sheets had an array of wells measuring 2 mm × 2 mm with a depth of 0.8 mm. Priory wad punches (Amazon.com) were used to punch discs from the sheets, which fit snugly into tissue culture plate wells. A nylon mesh (36 μm square pore openings, part number CMN-0035; Amazon.com) was bound to the open face of PDMS discs with silicone glue (Selleys Aquarium Safe). This mesh pore size is large enough to allow single cells through, but small enough to entrap aggregated cell *micro*-pellets within discrete microwells. Inserts were secured to tissue culture plastic with a small dab of silicone glue. Sterilisation was carried out with 70% ethanol. To ensure efficient contact of all surfaces and removal of any air bubbles, 4 mL of 70% ethanol solution was added to each well and centrifuged for 5 min at 2000×*g*. Plates were then submerged in 70% ethanol for 1 h at room temperature, followed by 3 washes with sterile phosphate-buffered saline (PBS; Thermo Fisher Scientific). PBS was left in wells overnight to elute residual ethanol. Before use, inserts were washed with a sterile 5% Pluronic solution (F-127 Pluronic; Sigma-Aldrich) in PBS. Pluronic renders the PDMS surface non-adhesive and promotes cell aggregation [27]. The plates were centrifuged for 5 min at 2000×*g* upon addition of Pluronic solution. Wells were rinsed three times with PBS, and the plates were ready for use in cell culture.

### Chondrogenic induction media

To induce chondrogenesis, BMSC were resuspended in chondrogenic medium composed of high glucose DMEM (HG-DMEM; Thermo Fisher Scientific), 1X GlutaMax (Thermo Fisher Scientific), 10 ng/mL transforming growth factor-beta 1 (TGF-β1, PeproTech), 100 nM dexamethasone (Sigma-Aldrich), 200 μM ascorbic acid 2-phosphate (Sigma-Aldrich), 100 μM sodium pyruvate (Thermo Fisher Scientific), 40 μg/mL L-proline (Sigma-Aldrich), 1% insulin-transferrin-selenium (ITS-X, Thermo Fisher Scientific), and 100 U/mL PenStrep (Thermo Fisher Scientific). In preliminary studies, we trialled constant PTH(1–34) (Sigma-Aldrich) at a concentration of 0, 1, 10, or 100 nM, captured microscopic images, performed histology, and live/dead viability staining (Thermo Fisher Scientific). We noted that continuously treated *micro*-pellets were reduced in size at all PTH concentrations, but appeared to have similar GAG staining (Alcian blue). Following this trial, all subsequent experiments were conducted with 2.5 nM PTH, as this concentration was used in previous work by Fischer et al. [23]. Cultures were maintained for 14 days, with a full medium exchange performed every 2 days. Three different PTH exposure protocols were compared: (1) control (chondrogenic medium with no PTH); (2) intermittent PTH with 2.5 nM PTH(1–34) added 6 h prior to media exchange, performed every 48 h; and (3) constant PTH supplementation with 2.5 nM PTH(1–34) replenished during each 48-h media exchange.

### Assembly of *Micro*-pellets within the *Microwell-Mesh* platform

To prepare plates for seeding, 1 mL of chondrogenic induction medium was added to each well, and *Microwell-mesh* plates were centrifuged for 5 min at 2000×*g* to eliminate any air bubbles from the microwells. Experiments were performed in 6-well plates, and each well was seeded with 1.2 × 10^6^ cells, yielding 5000 cells per microwell. BMSC were centrifuged for 5 min at 500×*g* to pellet the cells into microwells. Cultures were maintained at 2% O_2_ and 5% CO_2_ in a 37 °C incubator and media exchanged every other day (or 48 h). Our group previously showed that BMSC chondrogenesis was improved at 2% O_2_ [24, 25]. At the time of harvest, the mesh was removed from the *Microwell-mesh* disc with forceps and the *micro*-pellets were collected with a wide pipette tip. Aliquots of medium were collected at each medium exchange and stored at − 30 °C for later GAG quantification.

### Glycosaminoglycan and DNA quantification

*Micro*-pellets were digested overnight in papain (1.6 U/mL, Sigma-Aldrich) in a 60 °C water bath. Digests were vortexed briefly and used for GAG and DNA quantification. The 1,9-dimethylmethylene blue (DMMB, Sigma-Aldrich) assay was used to quantify GAG as described previously [25]. Chondroitin sulfate from shark cartilage (Sigma-Aldrich) was used to generate a standard curve. DNA content was determined using a Quant-iT PicoGreen dsDNA assay kit as per the manufacturer’s protocol (Thermo Fisher Scientific).

### Quantitative polymerase chain reaction

*Micro*-pellets were washed in PBS and stored in TRIzol (Thermo Fisher Scientific) at − 80 °C. RNA isolation was completed as per the manufacturer’s protocol followed by DNase I digest. RNA was quantified using a NanoDrop Lite spectrophotometer (Thermo Fisher Scientific). RNA (1000 ng) was reverse-transcribed using the SuperScript III First-Strand Synthesis System for qPCR (Thermo Fisher Scientific) as described by the manufacturer. The master mix included 2X SYBR Green PCR Master Mix (Applied Biosystems), 200 nM of the forward and reverse primers, RNase-free water, and 1 μL of sample cDNA. The reactions were run in triplicate using 5 μL per well in a 384-well plate inside a Viia7 Real-Time PCR System (Applied Biosystems). The initial cycle was 50 °C for 2 min and 95 °C for 10 min, followed by 40 cycles of 95 °C for 15 s and 60 °C for 1 min. A melt curve analysis was used to confirm the specificity of products. Gene expression data sets were analysed for statistically significant differences between conditions at each time using ANOVA. Primer set information is given in Table 1. Primers for *ACAN*, *COL1A1*, *COL2A1*, and *BGLAP/PB-OST* [28]; *RPLP0* and *COL10A1* [29]; *RUNX2* [30]; and *IHH* [31] were as previously published in the literature. Primers for *SOX9* were designed using Primer3Plus software [32]. *RPLP0* was found to be stable across conditions and was used to standardise relative expression of chondrogenic, osteogenic, and hypertrophy genes.

**Table 1:** Primers used for qPCR of human genes

Gene	Sequence (5′ to 3′)	Amplicon size (bp)
*ACAN*	F: TCGAGGACAGCGAGGCC R: TCGAGGGTGTAGCGTGTAGAGA	85
*COL1A1*	F: CAGCCGCTTCACCTACAGC R: TTTTGTATTCAATCACTGTCTTGCC	83
*COL2A1*	F: GGCAATAGCAGGTTCACGTACA R: CGATAACAGTCTTGCCCCACTT	79
*RPLP0*	F: TGTGGGCTCCAAGCAGATGCA R: GCAGCAGTTTCTCCAGAGCTGGG	137
*COL10A1*	F: ACTCCCAGCACGCAGAATCCA R: TGGGCCTTTTATGCCTGTGGGC	132
*RUNX2*	F: GGAGTGGACGAGGCAAGAGTTT R: AGCTTCTGTCTGTGCCTTCTGG	133
*SOX9*	F: ACTCCTCCTCCGGCATGAG R: GCTGCACGTCGGTTTTGG	102
*IHH*	F: ATGAAGGCAAGATCGCTCG R: GATAGCCAGCGAGTTCAGG	149
*BGLAP/PB-OST*	F: GAAGCCCAGCGGTGCA R: CACTACCTCGCTGCCCTCC	70

### Histology and immunohistochemistry

*Micro*-pellets were harvested at days 7 and 14 and fixed in 4% paraformaldehyde (PFA) for 15 min and frozen in Tissue-Tek OCT compound (Sakura Finetek). Samples were cryosectioned with a Leica Cryostat CM1850 (Leica) at 7 μm and collected onto poly-lysine-coated slides (Thermo Fisher Scientific) and stored in a freezer until further processing. The sectioned tissues were fixed for 15 min with 4% PFA and washed with PBS. Alcian blue staining was performed to visualise GAG present in the tissues. Sections were stained with 1% Alcian blue (Sigma-Aldrich) solution in 3% acetic acid (pH 2.5) for 30 min. Sections were rinsed with tap water and counterstained with Nuclear Fast Red for 5 min, rinsed with tap water, and mounted (CC/mount, Sigma-Aldrich) for imaging. Immunohistological staining was performed for collagen type I, type II, and type X. Sections were permeabilised for 5 min with 0.1% Triton X-100 and blocked for 30 min at room temperature (RT) with 10% normal goat serum (Invitrogen). Primary antibodies (Abcam) raised against collagen type I (1:800), type II (1:100), and type X (1:100) were diluted in 1% BSA/PBS-T (PBS-0.1% Tween-20), and sections were incubated with antibodies at 4 °C overnight. Sections were rinsed twice for 5 min with 0.025% Triton X-100/PBS. Slides were incubated with 0.3% H_2_O_2_ in 100% methanol for 15 min and rinsed twice with PBS. Sections were incubated for 60 min at room temperature with secondary antibodies, goat anti-rabbit IgG H&L (HRP; ab6721) or goat anti-mouse IgG H7L (HRP; ab97023; both 1:1000; Abcam), in 1% BSA/PBS, then washed twice with PBS. The DAB kit chromogen solution was applied for 8 min, and slides were rinsed thoroughly in water. Nuclei were stained with Nuclear Fast Red for 2 min and rinsed with water.

### Data collection and analysis

Statistical analysis was performed using GraphPad Prism. Data were analysed using two-way ANOVA, and statistical significance was determined using Tukey’s test. Experiments were carried out with 3 unique biological donors. Results are represented as mean ± SD, *n* = 4, *P* < 0.05 unless otherwise noted.

## Results

### Intermittent and constant PTH treatment reduced *Micro*-pellet size

**Fig. 2:**
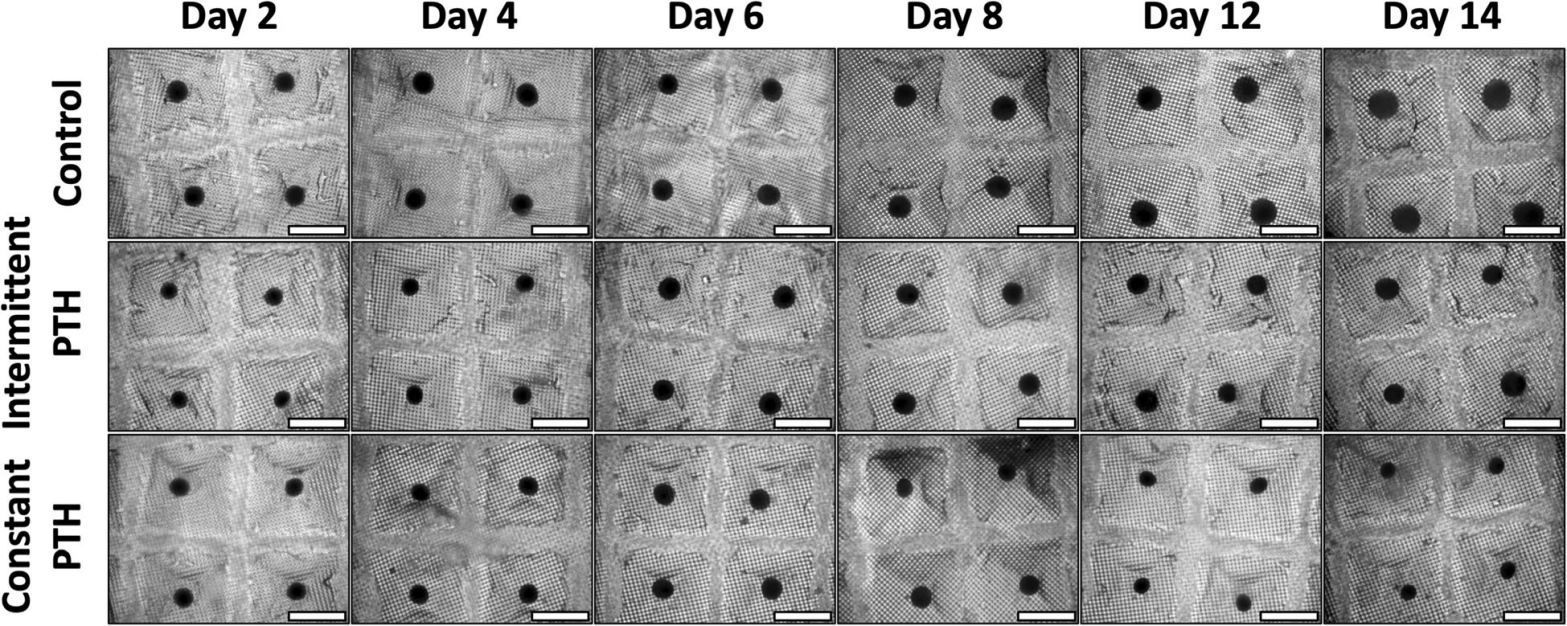
Microscope images of *micro*-pellets in the *microwell-mesh* cultured over 14 days. Scale bar, 1 mm. Replicate images from BMSC donors 2 and 3 are shown in Supplementary Figures 2 and 3, respectively

We initially tested *micro*-pellet induction cultures supplemented with constant PTH at concentrations of 0, 1, 10, and 100 nM. Microscopy imaging indicated that *micro*-pellet size was reduced with increasing concentrations of PTH; however, GAG staining was not affected and *micro*-pellets grown in continuous 2.5 nM PTH appeared viable (Supplementary Figure 1). Based on these data, subsequent experiments were completed with 2.5 nM PTH to maintain consistency with previous BMSC chondrogenic studies [23]. Following 14 days of chondrogenic induction culture, *micro*-pellets in the control (no PTH) condition increased in size over the culture period and were visibly larger than the intermittent and constant PTH treatment *micro*-pellets (Fig. 2). *Micro*-pellets exposed to intermittent PTH grew modestly in size over the 14-day culture, whereas *micro*-pellets in constant PTH culture grew until about 1 week and then appeared to decrease in size during the last week of culture.

### Intermittent and constant PTH inhibit glycosaminoglycan production

**Fig. 3:**
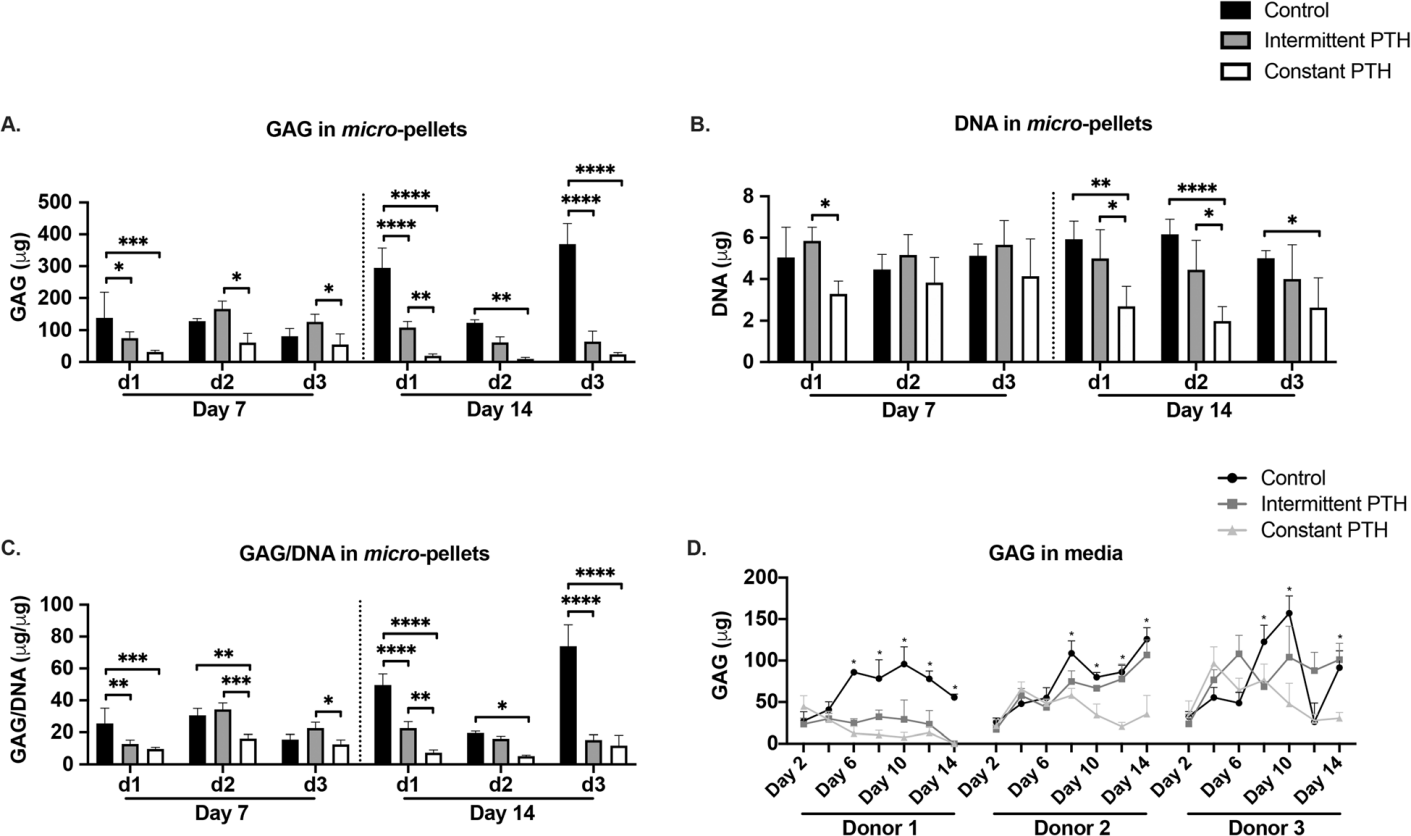
Quantification of GAG and DNA in *micro*-pellet cultures generated from three BMSC donors. **a** Quantities of GAG in *micro*-pellets at day 7 and day 14. **b** DNA quantities in *micro*-pellets at day 7 and day 14. **c** GAG normalised to DNA in *micro*-pellets. **d** Quantification of GAG secreted to the media by *micro*-pellets over a 14-day culture period. For **a**–**c**, data represent mean ± SD, *n* = 4, *P < 0.05, **P < 0.01, ***P < 0.001, and ****P < 0.0001. For **d**, data represent mean ± SD, *n* = 6, a single asterisk is used for all levels of significance when *P* < 0.05

We compared the GAG and DNA content of *micro*-pellets in control, intermittent PTH, and constant PTH chondrogenic cultures (Fig. 3). At days 7 and 14, the GAG content in constant PTH *micro*-pellets was significantly lower than in control and intermittent PTH *micro*-pellets (Fig. 3a). At day 14, both intermittent PTH and constant PTH *micro*-pellets had reduced GAG content when compared with control *micro*-pellets, and this was statistically significant for 2 of 3 donors (Fig. 3a). DNA quantities were similar across conditions at day 7, while constant PTH *micro*-pellets had significantly less DNA than the control group at day 14 (Fig. 3b). When GAG was normalised to DNA, GAG/DNA was reduced following both intermittent PTH and constant PTH treatment, relative to control (Fig. 3c). Additionally, less GAG was present in the medium in both intermittent PTH and constant PTH cultures, relative to control cultures (Fig. 3d).

### Intermittent and constant PTH both inhibit extracellular matrix production

**Fig. 4:**
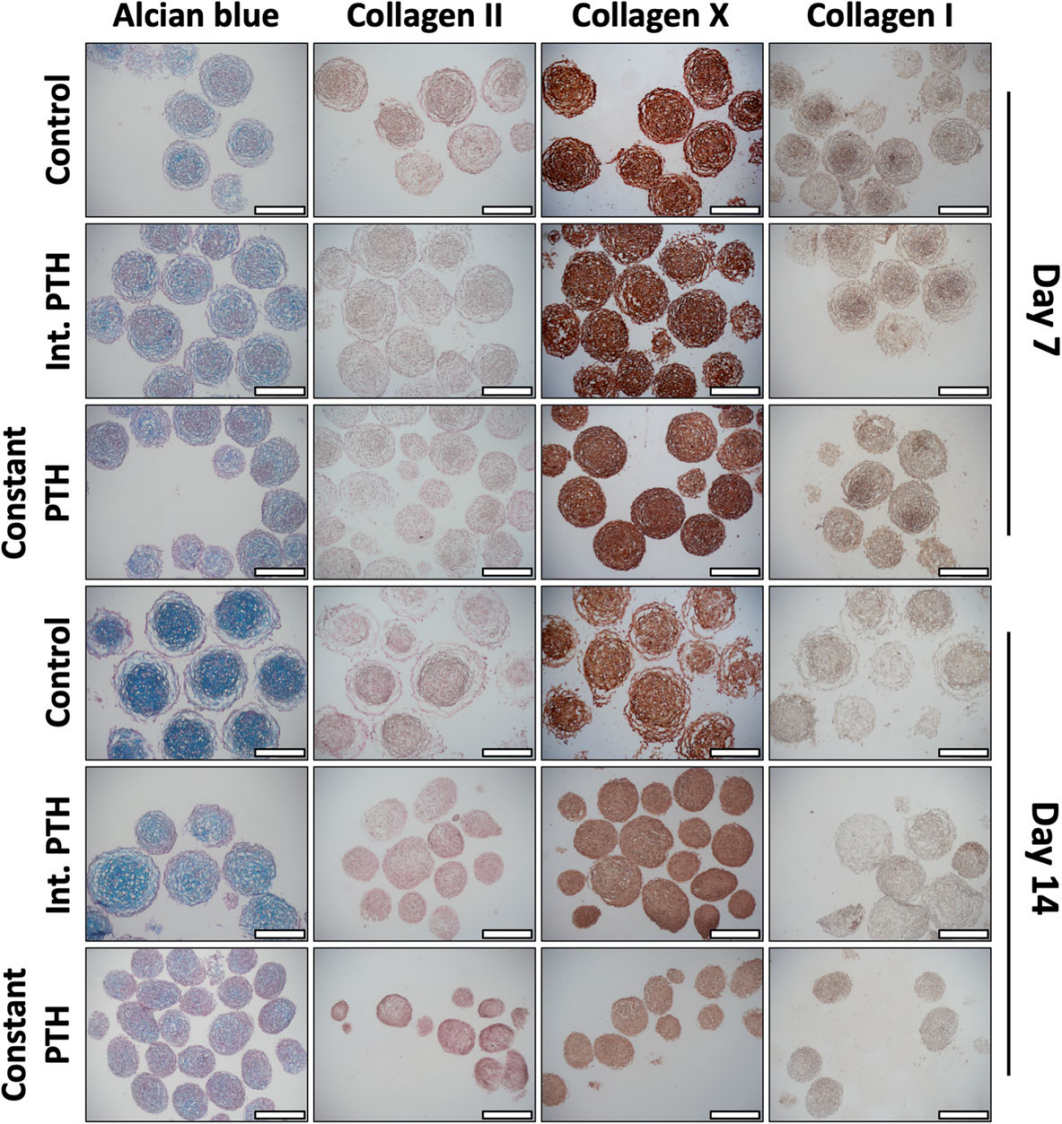
*Micro*-pellet sections stained for GAG (Alcian blue), collagen II, X, and I. Scale bar = 400 μm. Refer to Supplementary Figures 4 and 5 for replicate donor results

We analysed extracellular matrix makers associated with chondrogenesis (GAG and collagen II), hypertrophy (collagen X), and fibrogenesis (collagen I) histologically for control, intermittent PTH, and constant PTH conditions (Fig. 4). Alcian blue staining for GAG and immunohistochemistry staining for collagen II, X, and I were similar in *micro*-pellets from each condition on day 7, but notably reduced by constant PTH treatment on day 14 (Fig. 4). Neither intermittent PTH nor constant PTH improved BMSC chondrogenesis, relative to control, based on the staining of these markers.

### PTH decreased the expression of osteogenic, hypertrophic, and chondrogenic genes

**Fig. 5:**
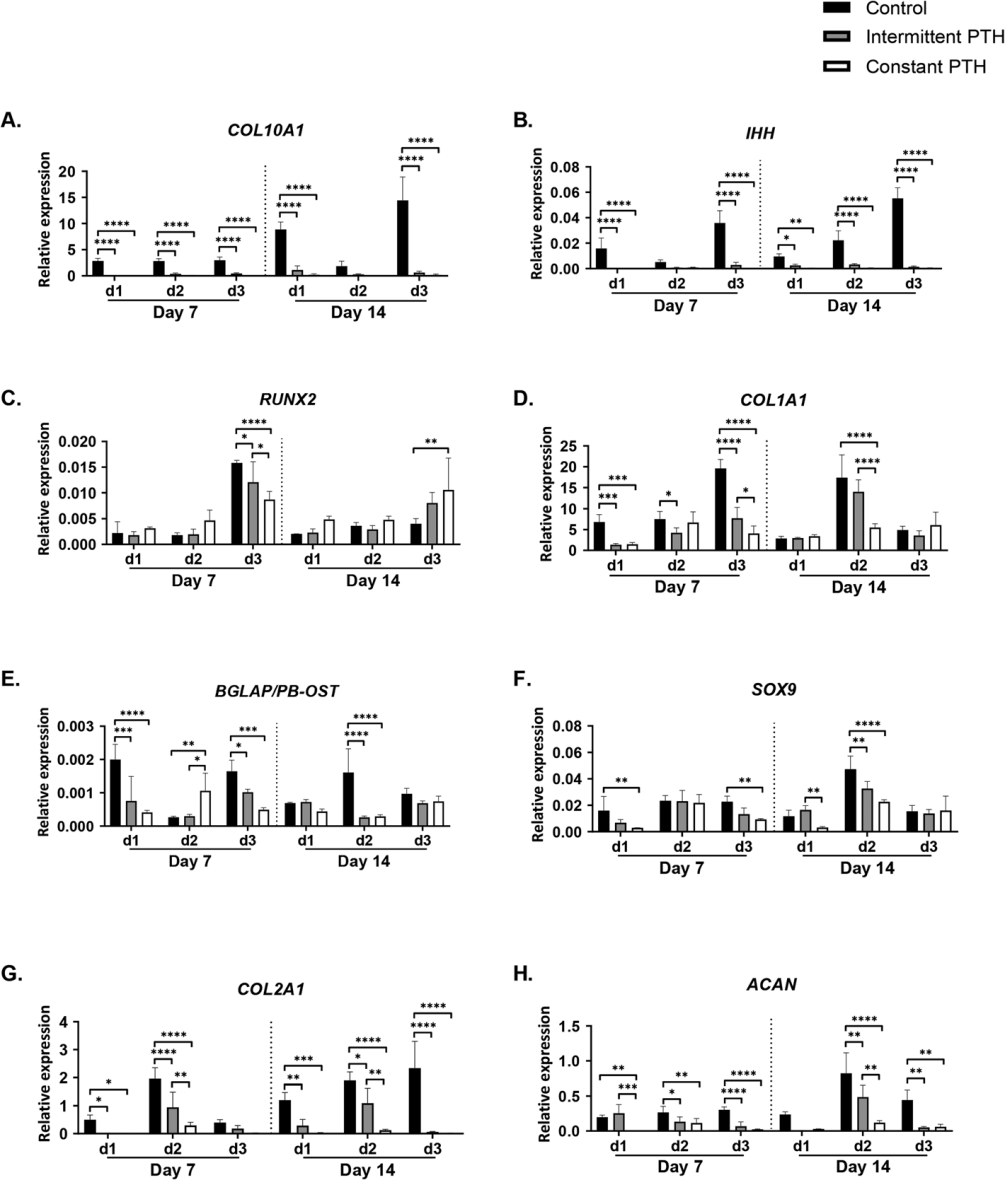
qPCR analysis of *micro*-pellets generated from three BMSC donors. Relative gene expression analysis of **a***COL10A1*, **b***IHH*, **c***RUNX2*, **d***COL1A1*, **e***BGLAP/PB-OST*, **f***SOX9*, **g***COL2A1*, and **h***ACAN*. *Micro*-pellets supplemented with constant PTH demonstrated a decrease in hypertrophic and osteogenic markers that was accompanied by a decrease in chondrogenic markers *SOX9*, *COL2A1*, and *ACAN* (plotted as mean ± SD, *n* = 4, *P < 0.05)

We assessed the expression of osteogenic, hypertrophic, and chondrogenic genes for control, intermittent PTH, and constant PTH conditions using qPCR (Fig. 5). PTH-treated *micro*-pellets had a decrease in hypertrophic marker expression (*COL10A1* and *IHH*, Fig. 5a, b) at day 7 and day 14, compared with control. *RUNX2*, a master regulator of osteogenesis, expression was similar across conditions, although in one donor they were reduced at day 7 and increased at day 14 (Fig. 5c). PTH supplementation resulted in significantly lower levels of osteogenic markers *COL1A1* and osteocalcin (*BGLAP/PB-OST*) in donors 1 and 3 at day 7, and donor 2 at day 14 (Fig. 5d, e). The expression of chondrogenic marker *SOX9* (Fig. 5f) was generally similar between groups or reduced in the presence of PTH, while *COL2A1* (Fig. 5g) and *ACAN* (Fig. 5h) were reduced by intermittent PTH or constant PTH, relative to control conditions without PTH. Overall, it appeared that both PTH conditions suppressed the undesirable expression of hypertrophic and osteogenic genes, as well as the desirable chondrogenic genes.

## Discussion

BMSC have shown potential as a cell source for cartilage tissue engineering. However, following BMSC chondrogenesis, hypertrophy occurs, yielding a bone-like tissue that is unsuitable for cartilage defect repair [2]. This hypertrophic process must be mitigated to allow the chondrocyte phenotype to persist, enabling these cells to produce and maintain a functional hyaline-like cartilage tissue. Many molecules have been investigated for their potential role in inhibiting hypertrophy (previously reviewed [2, 3]). PTH has previously been shown to inhibit the terminal differentiation of chondrocytes [5, 19], inhibit articular cartilage degeneration [33], and to promote the reversion of hypertrophic chondrocytes to a pre-hypertrophic proliferating phenotype [34]. PTH also reduced the expression of hypertrophy marker, collagen X, in BMSC from osteoarthritic patients [35]. Other studies suggest, however, that PTH inhibits type II collagen synthesis [4, 17, 18], an essential extracellular matrix component in articular cartilage. The utility of PTH in obstructing hypertrophy of BMSC cultured in chondrogenic induction medium in vitro remains unclear.

We posit that common large-diameter cartilage tissue models, which suffer large diffusion gradients and tissue heterogeneity, confound efforts to identify optimal BMSC chondrogenic induction protocols. In previous work, we demonstrated that it was possible to obtain more definitive insights into BMSC chondrogenesis, in vitro, if a more homogenous *micro*-pellet model system was used [24–26, 36]. In previous studies, it was reported that intermittent and constant PTH treatment of cells could result in profoundly alternate differentiation outcomes [18, 20–23]. In this study, we used a *micro*-pellet platform [24] with the aim of generating more definitive insights into how intermittent or constant PTH treatment influenced BMSC chondrogenesis and hypertrophy. We found that *micro*-pellets in chondrogenic differentiation media treated with intermittent PTH and constant PTH experienced a reduction in *micro*-pellet size by day 14, relative to control cultures, with the most drastic decrease observed with constant PTH. Based on histological evaluation, constant PTH showed a negative effect on chondrogenesis based on reduced staining of GAG and collagen II by day 14, while intermittent PTH appeared to have a lesser effect on these markers. A reduction in GAG production with either PTH treatment was also confirmed with GAG quantification assays. Based on gene expression, we found that both PTH treatments decreased the expression of hypertrophy markers *COL10A1* and *IHH*, as well as osteogenic markers *COL1A1* and osteocalcin (*BGLAP/PB-OST*). However, chondrogenic marker, *COL2A1* and *ACAN*, expression also decreased. The expression of osteogenic transcription factor, *RUNX2*, and chondrogenic transcription factor, *SOX9*, was not drastically changed by PTH treatment conditions.

In Fischer et al. [23], prolonged exposure to PTHrP resulted in reduced cell proliferation at day 14. We similarly observe that constant PTH resulted in reduced DNA quantities compared to those found in control and intermittent PTH *micro*-pellets at day 14. To rule out cytotoxicity, we performed a live/dead assay on control and constant PTH *micro*-pellets. Both had strong green staining (live) with negligible red staining (dead), suggesting that PTH was not cytotoxic in our studies.

Unlike constant PTH, Fischer et al. [23] found evidence that intermittent PTHrP treatment might represent a potential means to improve chondrogenesis of BMSC. By contrast, the outcomes of our study do not support the use of intermittent or constant PTH treatment for improving BMSC chondrogenesis or inhibiting hypertrophy. While there are some differences in our culture methods [23], this study represents the closest study to our own, allowing for some comparison to be made. In the previous study, GAG and DNA production was increased with intermittent PTHrP treatment [23], whereas in our current study, these parameters were not improved. In the previous study, with intermittent PTH, the expression of *COL2A1* was increased, *COL10A1* was unchanged, and *IHH* was decreased [23], whereas in our study, we observed a downregulation of all three genes, amongst other genes. Like in our study, the previous study [23] observed a striking reduction in pellet growth and *COL2A1* and *COL10A1* expression in constant PTH conditions. There are two major differences between our studies, the first being the number of cells used to generate chondrogenic pellets: 5 × 10^5^ cells per pellet in the previous study [23] and 5000 cells per *micro*-pellet in our study. The second was the duration of chondrogenic culture (6 weeks in the previous study [23] and 2 weeks in our study). In previous work, we demonstrated that pellets formed from 2 × 10^5^ cells each suffer profound gradients and yield heterogenous tissues [25, 26]. In large-diameter pellets, differentiation appears to occur first at the peripheral edge of the pellet, with differentiation in the core being delayed, likely due to metabolite limitation in large-diameter tissues. By contrast, small-diameter *micro*-pellets appear to be relatively synchronised in differentiation across their diameter. As a result of this size difference, our *micro*-pellet study likely represents a more sensitive assay for BMSC chondrogenic response to PTH. Because of the large size of the chondrogenic pellets used in the previous study (5 × 10^5^ cells per pellet [23]), it is likely that cellular response to PTHrP was muted within the core of the tissues, while being able to elicit a response by the cells near the surface of the pellet. The important influence of gradients on PTH signalling has been discussed in the tissue engineering context previously [37]. This is consistent with the authors’ observations, where there was a strong response at the surface of their pellets, and a muted response at the core [23]. In this case, averaged over a large pellet and a 6-week culture, they observed better chondrogenic outcomes with intermittent PTH. Conceptually, our *micro*-pellet is representative of the surface of their large pellet, and this thin and homogenous tissue did not benefit from similar intermittent PTH treatment.

It is possible that further optimisation of PTH administration in chondrogenic cultures may be required to apply this molecule in a meaningful way to improve chondrogenesis. PTH has been reported to induce early chondrogenesis while suppressing terminal differentiation of chondrocytes [19]. We observed that *micro*-pellets in the intermittent PTH condition grew in size in the first week of culture and then stopped increasing in size over the second week of culture, suggesting that BMSC differentiation stage may be an important factor to consider. It is possible that intermittent PTH has an anabolic effect on chondrogenesis in more primitive BMSC, which ceases, or becomes catabolic with extended PTH treatment, as the cells become more differentiated. Previous studies have reported that PTH exerts its function in osteogenesis by enhancing BMP signalling [6, 38]. Others have reported that antagonising BMP signalling can obstruct hypertrophy during BMSC chondrogenesis [39]. Using a combination of early intermittent PTH with other strategies, like BMP2 inhibition, may lead to improved protocols for BMSC chondrogenesis, but this will have to be trialled.

## Conclusion

In our hands, up to 1 week of intermittent PTH treatment suppressed BMSC hypertrophic gene expression in chondrogenic cultures, but this benefit was countered by its hindering effect on chondrogenesis by 2 weeks of culture. It is possible that PTH may have an anti-hypertrophic effect on more primitive BMSC and a catabolic effect on BMSC as they become increasingly differentiated. To apply intermittent PTH in a useful manner in BMSC chondrogenic culture, a better understanding of its molecular mechanism in these cells during differentiation would be beneficial. Furthermore, combining early, short-term intermittent PTH treatment with other strategies, such as BMP signalling inhibition, could result in more optimal BMSC chondrogenic protocols.

## Supplementary information

**Additional file 1: Supplementary Figure 1.** Concentration and live/dead assays of *micro*-pellets. *Micro*-pellets were treated with 1, 10 or 100 nM PTH or no PTH (control). A) Microscope images of *micro*-pellets within the microwell mesh at Day 14 of culture. Scale bar = 1 mm. B) Alcian blue staining of Day 14 *micro*-pellet sections with Nuclear Fast Red as a counterstain. Scale bar = 400 μm. C) Tissues were stained with LIVE/DEAD viability stain as per the manufacturer’s instructions (Thermo Fischer Scientific). LIVE/DEAD stain of control and PTH-treated (2.5 nM) *micro*-pellets at Day 14. Calcein-AM (green/live) and propidium iodide (red/dead) demonstrate relative viability in cultures without (control) or with PTH (2.5 nM) . Scale bar = 200 μm.

**Additional file 2: Supplementary Figure 2.** Microscope images of donor 2 *micro*-pellets within the *microwell-mesh*. Scale bar = 1 mm.

**Additional file 3: Supplementary Figure 3.** Microscope images of donor 3 *micro*-pellets within the *microwell-mesh*. Scale bar = 1 mm.

**Additional file 4: Supplementary Figure 4.** Alcian blue and collagen II, X and I staining of donor 2 *micro*-pellets. Scale bar = 400 μm.

**Additional file 5: Supplementary Figure 5.** Alcian blue and collagen II, X and I staining of donor 3 *micro*-pellets. Scale bar = 400 μm.

## Data Availability

Supporting data can be obtained from the corresponding author.

## Abbreviations

ACAN: Aggrecan
BGLAP/PB-OST: Osteocalcin
BMSC: Bone marrow-derived stromal cells
COL1A1: Collagen type I alpha 1 chain
COL2A1: Collagen type II alpha 1 chain
RPLP0: 60S acidic ribosomal protein P0
COL10A1: Collagen type X alpha 1 chain
DMMB: 1,9-Dimethylmethylene blue
DNA: Deoxyribonucleic acid
FBS: Fetal bovine serum
FGF-1: Fibroblast growth factor-1
GAG: Glycosaminoglycan
HG-DMEM: High glucose DMEM
ITS-X: Insulin-transferrin-selenium
LG-DMEM: Low glucose Dulbecco’s modified Eagle’s medium
PBS: Phosphate-buffered saline
PDMS: Polydimethylsiloxane
PenStrep: Penicillin/streptomycin
PTH: Parathyroid hormone
PTH1R: PTHrP receptor 1
qRT-PCR: Quantitative real-time polymerase chain reaction
RNA: Ribonucleic acid
RUNX2: Runt-related transcription factor 2
SOX9: SRY-Box transcription factor 9
IHH: Indian hedgehog
ssDNA: Single-stranded deoxyribonucleic acid
TGF-β1: Transforming growth factor-beta 1
