# Supplementary figures and images for: Intermittent parathyroid hormone (1–34) supplementation of bone marrow stromal cell cultures may inhibit hypertrophy, but at the expense of chondrogenesis
Source: Stem Cell Res Ther. 2020 Jul 29;11:321. doi: 10.1186/s13287-020-01820-6 (PMC7389809; doi:10.1186/s13287-020-01820-6)

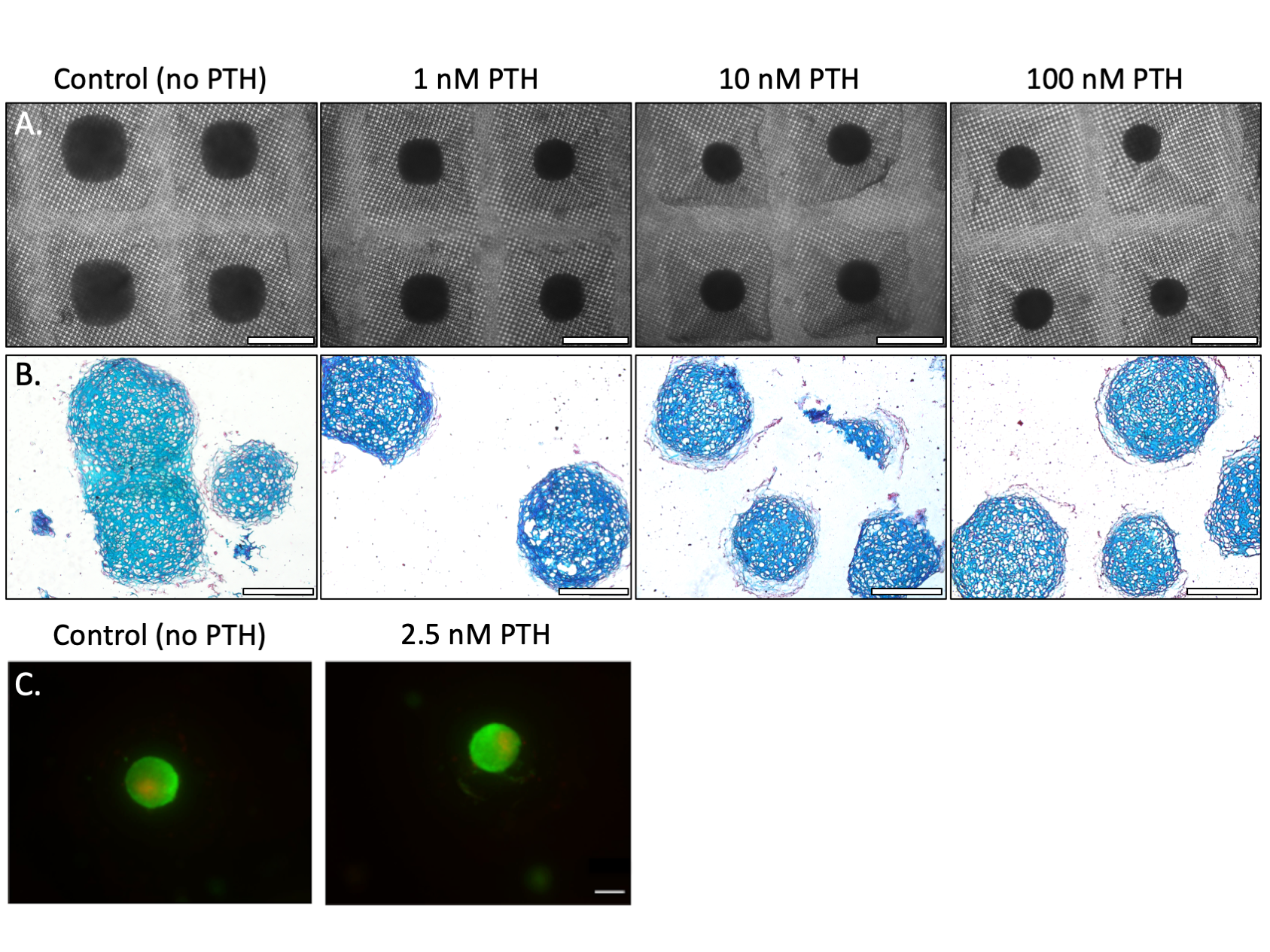

Supplement: Supplementary file 1 — Additional file 1: Supplementary Figure 1. Concentration and live/dead assays of micro-pellets. Micro-pellets were treated with 1, 10 or 100 nM PTH or no PTH (control). A) Microscope images of micro-pellets within the microwell mesh at Day 14 of culture. Scale bar = 1 mm. B) Alcian blue staining of Day 14 micro-pellet sections with Nuclear Fast Red as a counterstain. Scale bar = 400 μm. C) Tissues were stained with LIVE/DEAD viability stain as per the manufacturer’s instructions (Thermo Fischer Scientific). LIVE/DEAD stain of control and PTH-treated (2.5 nM) micro-pellets at Day 14. Calcein-AM (green/live) and propidium iodide (red/dead) demonstrate relative viability in cultures without (control) or with PTH (2.5 nM) . Scale bar = 200 μm. [file 13287_2020_1820_MOESM1_ESM.tiff]

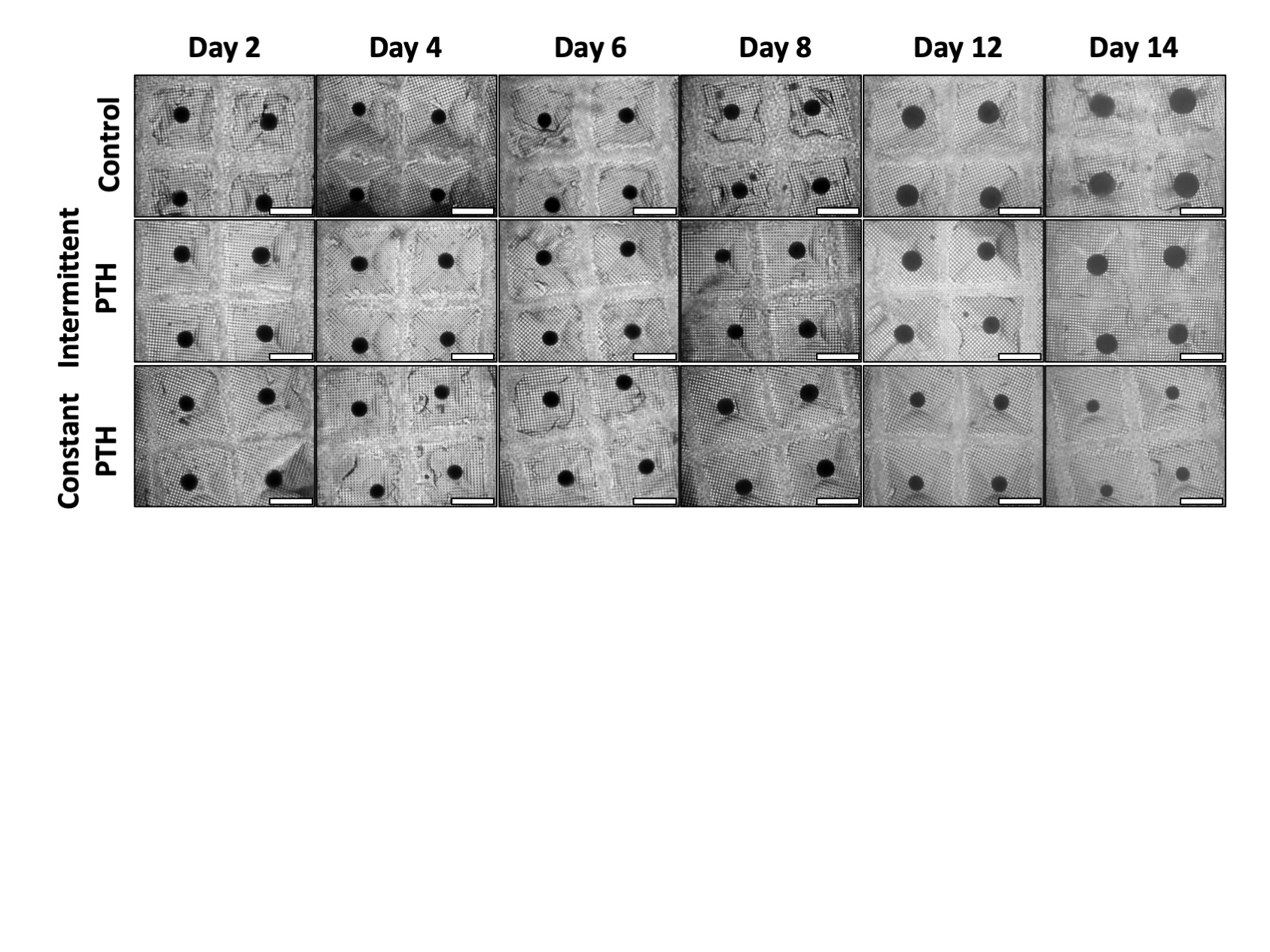

Supplement: Supplementary file 2 — Additional file 2: Supplementary Figure 2. Microscope images of donor 2 micro-pellets within the microwell-mesh. Scale bar = 1 mm. [file 13287_2020_1820_MOESM2_ESM.jpg]

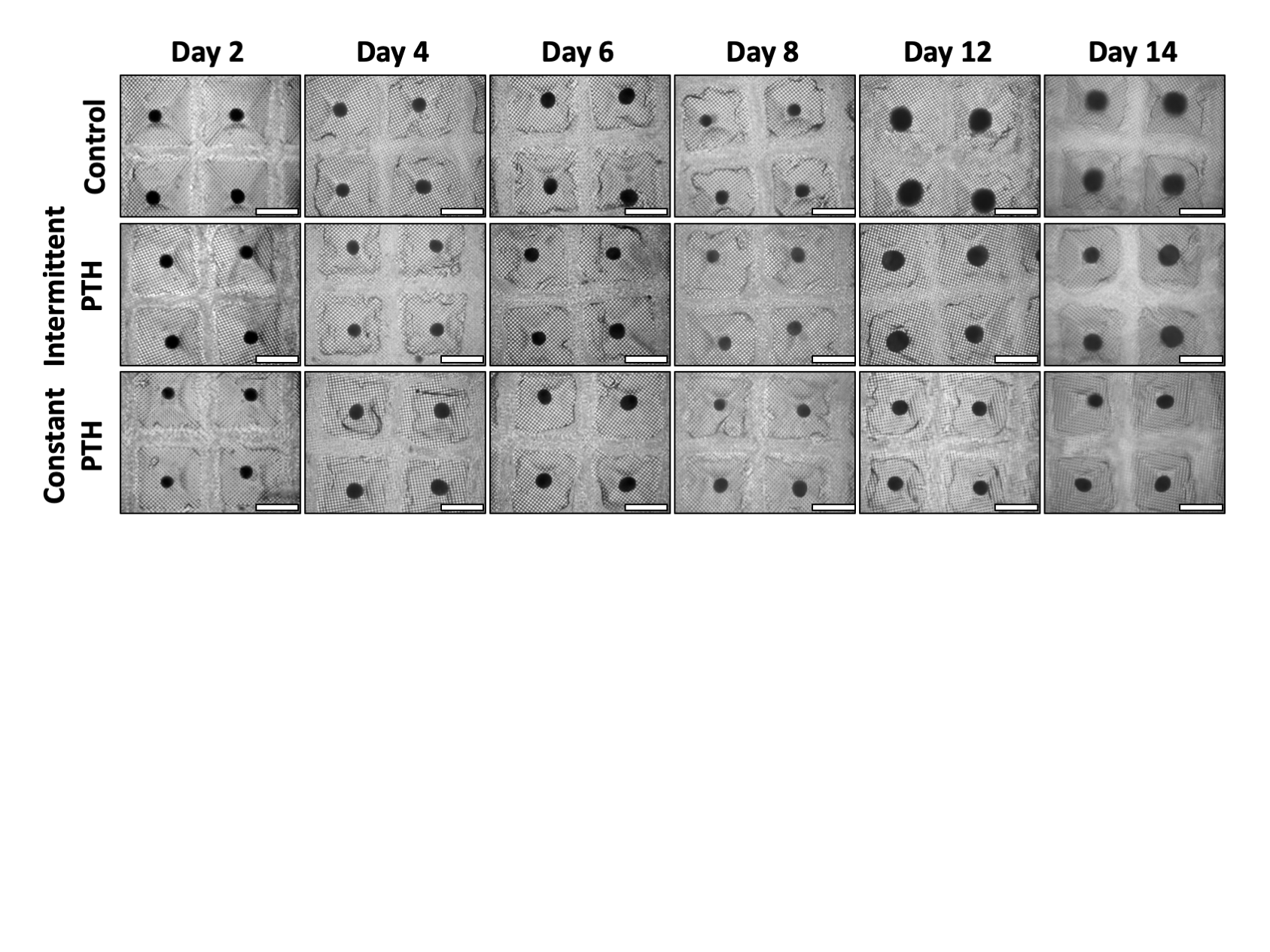

Supplement: Supplementary file 3 — Additional file 3: Supplementary Figure 3. Microscope images of donor 3 micro-pellets within the microwell-mesh. Scale bar = 1 mm. [file 13287_2020_1820_MOESM3_ESM.tiff]

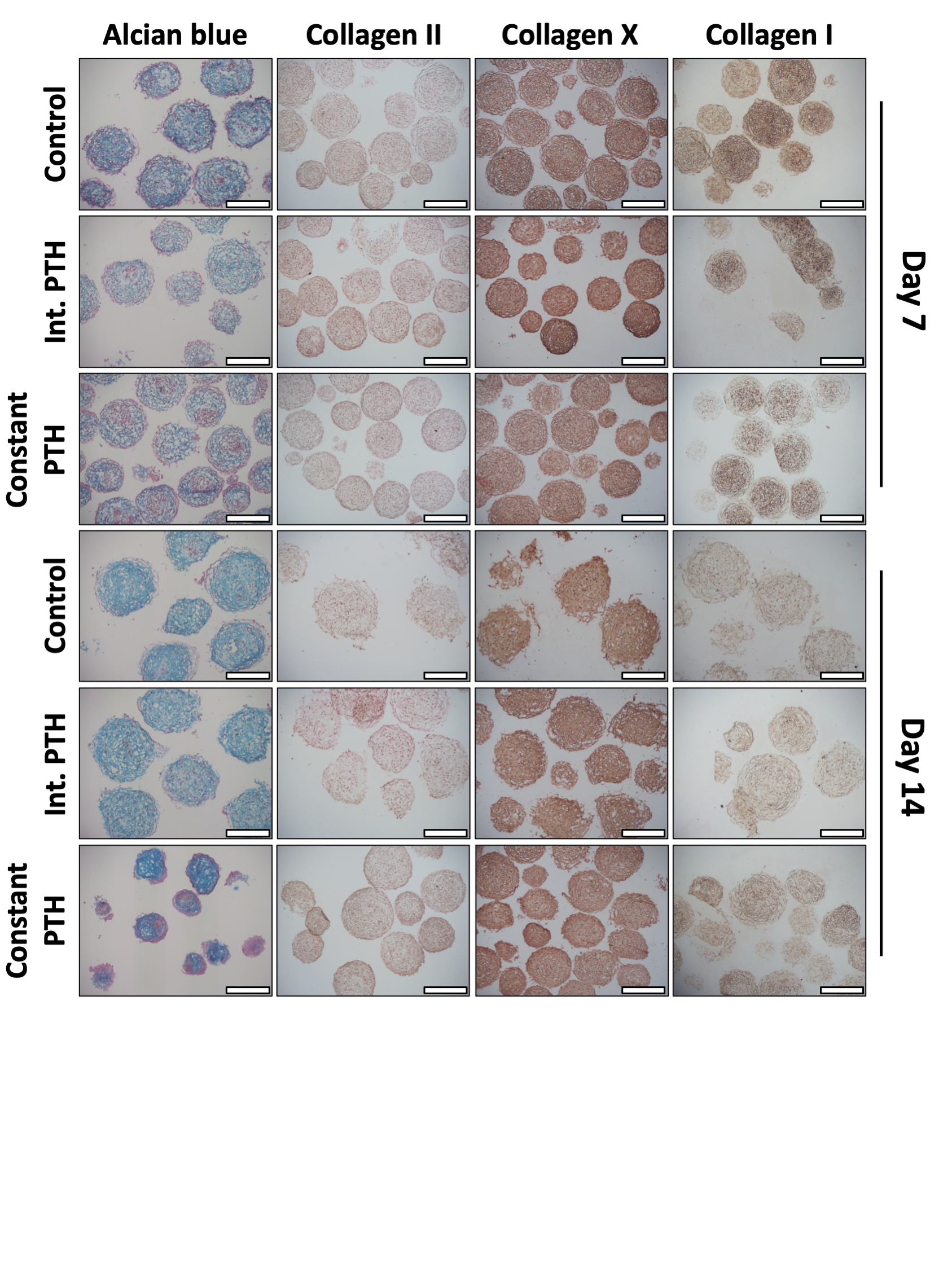

Supplement: Supplementary file 4 — Additional file 4: Supplementary Figure 4. Alcian blue and collagen II, X and I staining of donor 2 micro-pellets. Scale bar = 400 μm. [file 13287_2020_1820_MOESM4_ESM.tiff]

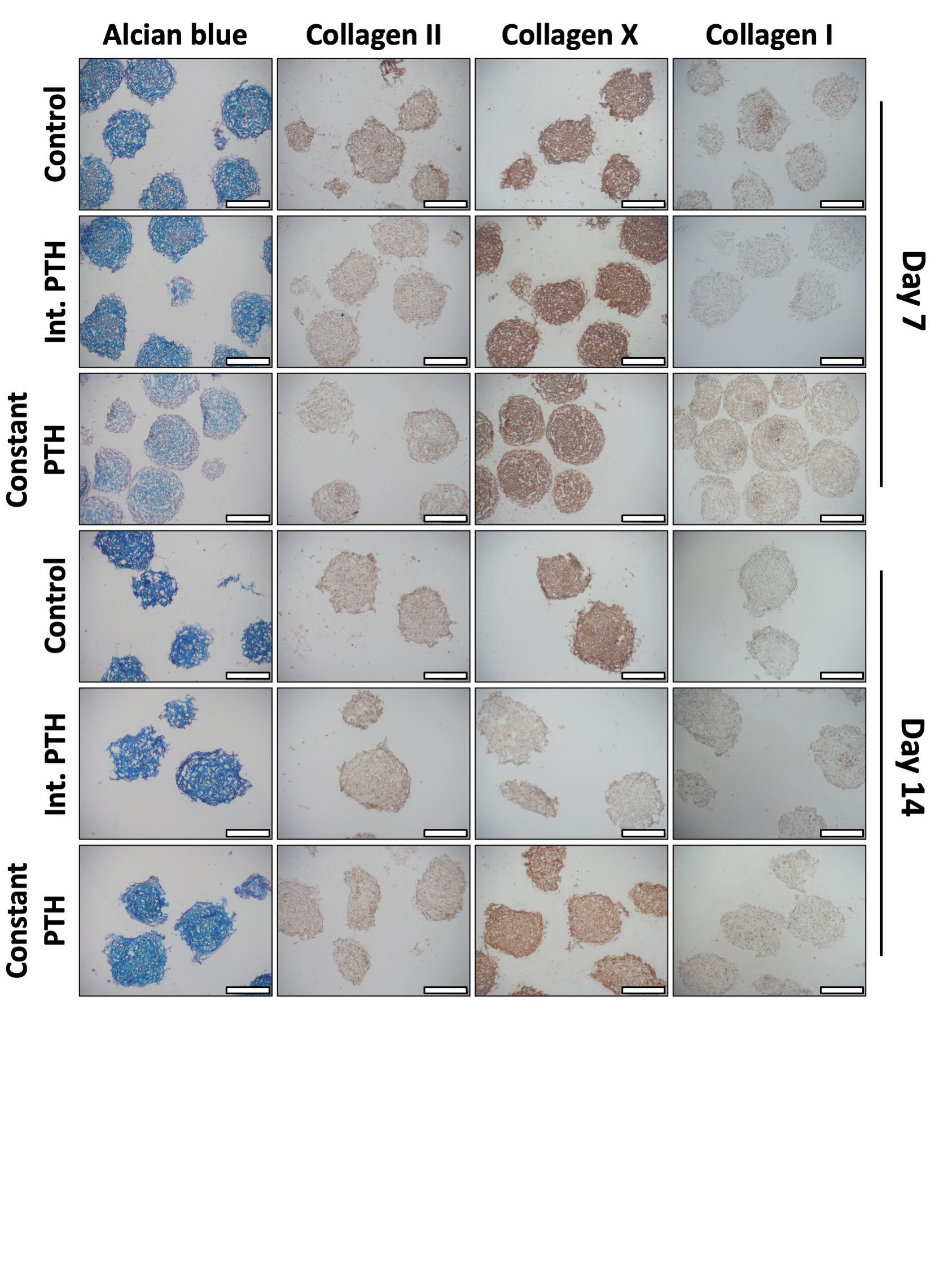

Supplement: Supplementary file 5 — Additional file 5: Supplementary Figure 5. Alcian blue and collagen II, X and I staining of donor 3 micro-pellets. Scale bar = 400 μm. [file 13287_2020_1820_MOESM5_ESM.tiff]
